# Arrhythmia incidence and risk factors in critically ill patients

**DOI:** 10.1186/cc14236

**Published:** 2015-03-16

**Authors:** G Tasdemir, N Kelebek Girgin, A Aydin Kaderli, E Cizmeci, R Iscimen, F Kahveci, A Aydinlar

**Affiliations:** 1 Uludag University Medical Faculty, Bursa, Turkey

## Introduction

Cardiac arrhythmias may be observed at any time during the ICU stay. The prognosis may suffer due to these arrhythmias. In our study, we aimed to evaluate incidence and risk factors of arrhythmias occurring in patients in the ICU.

## Methods

Patients treated in the ICU were included in the study if they fulfilled the following: age >18, no cardiac valvular disease, no cardiac surgery in the recent 6-month period, no history of myocardial infarction (MI), need for mechanical ventilation, and one or more organ failure. Demographic, hemodynamic and laboratory parameters, APACHE II score, presence of sepsis, acute renal failure, MI, and VIP during the ICU stay were recorded. Therapies used for arrhythmia and response to therapies were also recorded.

## Results

Two hundred and fourteen patients were included in the study. Twenty-six percent (*n *= 56) of patients had arrhythmias. Incidence was higher in females (*P *= 0.045). Average age of arrhythmic patients was 69 (19 to 86), and they were older than nonarrhythmic patients (*P *< 0.001). APACHE II scores were higher in arrhythmic patients (*P *= 0.001). Admission to the ICU with cerebrovascular event (CVE) and trauma was related to arrhythmia (*P *= 0.021, *P *= 0.032, respectively). There was a significant relationship between VIP and sepsis presence (*P *< 0.001, *P *< 0.001). Atrial fibrillation was the most frequent type of arrhythmia (53%), and the most frequently used medication was diltiazem (28.5%). The patients who had arrhythmias had a longer ICU stay (*P *= 0.021). The mortality rate for all patients was 48.1%. There was no statistically significant relationship between arrhythmia and mortality (*P >*0.05).

## Conclusion

Older age, higher APACHE II scores, trauma, CVE, VIP and sepsis increases arrhythmia risk in critically ill patients. Atrial fibrillation is most common and the most preferred treatment for all arrhythmias is diltiazem.
